# Reengineering of cancer cell surface charges can modulate cell migration†

**DOI:** 10.1039/d2cc00402j

**Published:** 2022-04-06

**Authors:** Mattia Ghirardello, Radhe Shyam, M. Carmen Galan

**Affiliations:** School of Chemistry, University of Bristol, Cantock's Close BS8 1TS Bristol UK m.c.galan@bristol.ac.uk https://www.galanresearch.com

## Abstract

The ability to modulate the cell surface structure provides a powerful tool to understand fundamental processes and also to elicit desired cellular responses. Here we report the development of a new class of ‘clickable labels’ to reengineer the cell surface charges of live cells. The method relies on the use of metabolic oligosaccharide engineering (MOE) combined with chemo selective labeling of cell surface azido-containing sialic acids with dibenzocyclooctyne (DBCO) ionic-probes. Using this strategy, we demonstrate that reducing the negative charge induced by the overexpression of cell surface sialic acids in cancer cells leads to a reduction in cell migration without affecting drug supceptibility.

## Abstract 2

Reducing the negative charges induced by the overexpression of cell surface sialic acids using cationic clickable labels leads to a reduction in cancer cell migration without affecting drug supceptibility.

## Graphical abstract

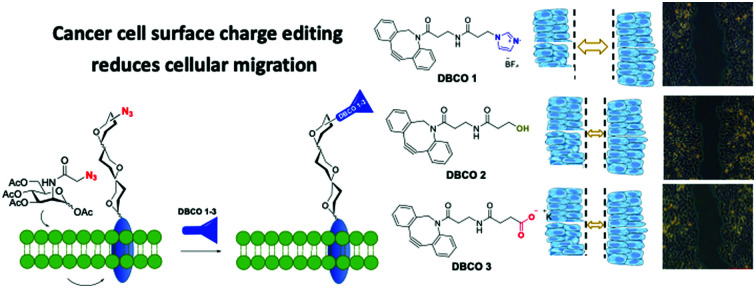

Cell adhesion and migration is essential in cell communication and regulation processes. As such, it plays fundamental roles in many biological processes such as embryogenesis, haematopoiesis, inflammation, immune responses and metastasis.^1,2^ Thus, the biochemical and mechanical interactions between cells and their extracellular matrix (ECM) influence their behaviour and ultimately their role and function.^2–4^ Cancer metastasis entails the relocation of malignant cells from a primary tumour site to distant organs, creating new tumour lesions. It is the key cause of failure in cancer therapies and is the primary cause of death in most cancer patients.^5^ To move from the primary tumour site, cancer cells have to change their adhesion properties increasing motility and invasiveness capabilities.^6^ The whole metastatic process consists of a series of biological events involving multiple biochemical and physical interactions between cancer cells with the ECM.^7–9^ However, despite the many advances in cancer research, the mechanisms behind this process are still not fully understood due to the complexity of the system.^6^

Altered glycosylation is one of the hallmarks of cancer,^10^ the abundant and aberrant overexpression of sialic acid-terminated glycosides on the cell surface has been correlated with immunosuppression, cell motility, cancer progression and metastasis.^11–16^ Evidence suggests that sialic acid-binding receptors found in immune cells, such as Siglecs and Selectins, are exploited by hypersialylated cancer cells to induce immunosuppression and to modulate key immune cell types in the tumour that are responsible for maintaining the appropriate inflammatory environment.^17–19^ For instance, it has been shown that hypersialylation may augment colon tumour progression by altering cell preference for certain extracellular matrix milieus, as well as by stimulating cell motility.^15^

Sialic acid is a negatively charged nine-carbon atom monosaccharide featuring a carboxylic acid group at the anomeric carbon. Thus, the aberrant overexpression of sialylated glycans in cancer cells leads to a more negatively charged cell surface when compared to normal cells.^16^ Moreover, many of the major functions in cells and organs of the human body are controlled by ionic currents, electric fields, ion flow, and voltage gradients produced by ion channels and pumps, which are also responsible for cell proliferation, migration, and differentiation processes.^20^ On this basis, we hypothesize that cellular migration could be modulated by modifying the cell surface charges and in particular those resulting from the overexpression of cell surface sialic acid residues.

Metabolic oligosaccharide engineering (MOE) is a strategy that allows the incorporation of modified sugar residues bearing an unnatural chemical reporter onto glycoproteins.^21,22^ The labelled sugars are converted by the cell biosynthetic machinery into activated nucleotide sugars that are transported into the Golgi and then transferred to glycoconjugates destined for secretion, delivery to cellular compartments or presentation on the cell surface. On this context, a number of metabolic glycan reporters have been successfully used to highjack glycan biosynthesis, chemically modify cell surfaces, probe intracellular metabolic flux inside cells, and to identify specific glycoprotein subtypes from the proteome.^21,23–28^*N*-Azidoacetylmannosamine (Man*N*Az) is most commonly used to visualize sialic acid-containing glycoproteins in living cells.^29^ Man*N*Az utilizes an azide functional group as the chemical reporter, which upon being metabolized, can be selectively derivatized using the Staudinger ligation,^30^ the Cu(i) catalyzed^31^ or the strain-promoted^32–35^ azide–alkyne [3+2] cycloaddition.

Previously in our lab, we described the use of imidazolium tagged-mannosamine derivatives as a chemical reporter that could be metabolically incorporated into cell-surface sialic acids.^22^ However, low levels of cationic-labelled cell surface sialic acids were detected as determined by the electrokinetic potential (zeta potential) and fluorescent measurements, hampering its use for our proposed study of the effect on cell surface charge editing. In order to evaluate the effect of modifying the ionic charges of sialylated cell surfaces, a higher degree of cell surface expressed chemical reporters is required. Thus, we proposed that chemo-selective modification of cell surface azido-containing sialic acids with a suitably “clickable” probe would be more efficient. To that end, the strain-promoted azide–alkyne cycloaddition (SPAAC) reaction employing tetracetylated *N*-Azido-acetylmannosamine (Ac_4_Man*N*Az) as the MOE-chemical reporter^25^ and a suitably functionalized DBCO probe was chosen. To that end, cationic DBCO 1, neutral DBCO 2 and anionic DBCO 3 were prepared starting from commercial DBCO-NH_2_4 in one step and in 46–60% yields after HPLC isolation. EDC/NHS promoted amide coupling of 4 with carboxylic-Imidazolium (ITag) 5 furnished 1, while ring opening of succinic anhydride or β-propiolactone with 4 gave access to 2 and 3, respectively (Scheme 1).

**Scheme 1:**
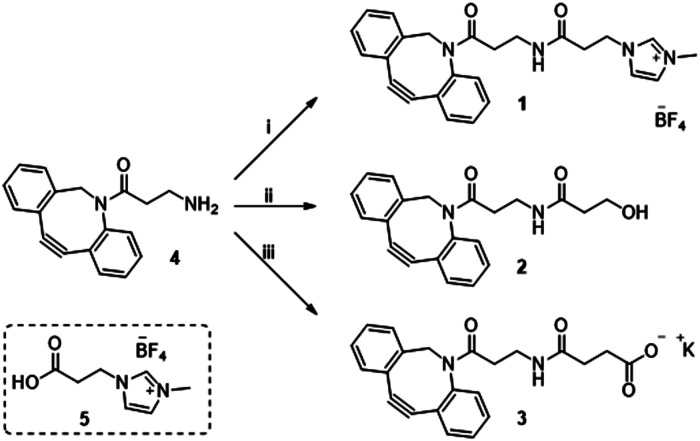
*Reagent and conditions*: (i) 5, EDC·HCl, NHS, CH_3_CN, 21 h, rt 46%; (ii) β-propiolactone, toluene, 24 h, rt 60%; (iii) succinic anhydride, DCM, 16 h, rt then K_2_CO_3_, MeOH/H_2_O, 2 h, rt 53%.

To determine optimal dosage and cell cytotoxicity of the new DBCO-labels, cervical cancer (HeLa) cells and human umbilical vein (EA·hy926) cells were exposed for 1 hour to a range of concentrations of DBCO-probes 1–3 (from 12.5 μM to 100 μM). Metabolic competence was then assessed using Alamar Blue and compared to untreated controls. It was found that 25 μM for all the probes and across the two cell lines was optimal to maintain close to 100% viability, while at higher concentrations viability dropped to 50–65% (see details and Fig. S7 in ESI†).

Next, we evaluated the ability of our DBCO-probes to label cell surface sialoglycans expressing the N_3_ reporter by using a competitive fluorometric assay with commercial DBCO-Cy5 dye. Breast cancer MDA-MB231 cells, HeLa cells and EA·hy926 cells were used as model systems. Cells previously treated with 25 μM Ac_4_Man*N*Az for 3 days to metabolically express cell surface *N*-azidoacetylneuraminic acid where then exposed to 25 μM of DBCO-probes 1–3, respectively, for 1 hour at 37 °C, followed by a 2nd labelling step with DBCO-Cy5 before measuring the fluorescence (Fig. 1, group A, and Tables S1–S3, ESI†). Results were then compared to control cells, *e.g.* cells that had been treated with Ac_4_Man*N*Az and underwent the 2nd labelling step with fluorescent DBCO-Cy5 alone (control M) and untreated cells exposed to DBCO-Cy5 (control). As expected, the fluorescence of cells that had been labelled with DBCO-probes 1–3 prior to DBCO-Cy5 exposure was significantly reduced. Additionally, to assess non-specific labelling, cells grown in the absence of Ac_4_Man*N*Az were also exposed to the same 2-step labelling procedures and showed low levels of background fluorescence (Fig. 1, group B). These results confirm the effective labelling with the novel DBCO-probes (>70% of all azido motifs were reacted when compared with DBCO-Cy5 labelling alone (control M)).

**Fig. 1:**
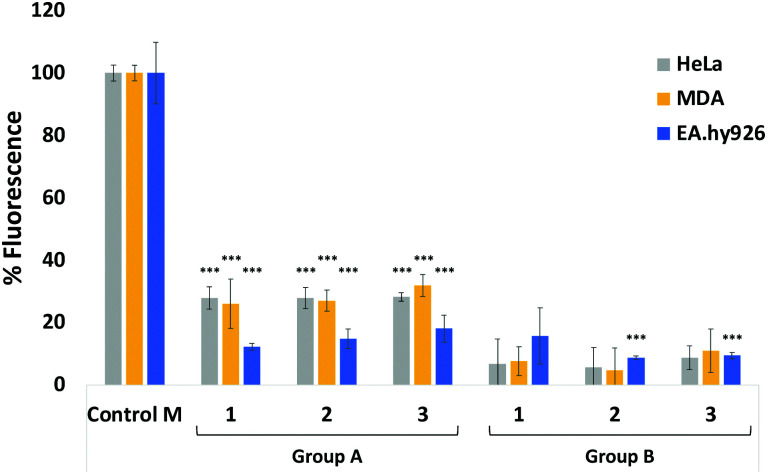
Relative fluorescence % of HeLa, MDA and EA·hy926 cells which have been incubated with Ac_4_Man*N*Az (25 μM) and labelled with DBCO-Cy5 (Control M); or cells incubated with Ac_4_Man*N*Az (Group A) or untreated cells (Group B) which were labelled with DBCO-probes 1–3 and DBCO-Cy5 and compare to control M set to 100%. Data shown after background fluorescence subtraction from native cells exposed to DBCO-Cy5 treatment (blank control). not significant (ns) *p* > 0.05, ***: *p* < 0.001.

Sialic acid expressed on the cell surface is a major contributor to the net negative charge on the surface of mammalian cells. To confirm the impact of the clickable tags on cell surface charges, the electrokinetic potential (Zeta potential) of HeLa, MDA and EA·hy926 cells which had been treated with 25 μM Ac_4_Man*N*Az and further labelled with 25 μM of either cationic DBCO-1, neutral DBCO-2 or anionic DBCO-3, as described before, was measured and compared to that of controls *e.g.* untreated cells, cells treated with Ac_4_Man*N*Az (control M) and untreated cells exposed to DBCO-1 (Fig. 2 and Tables S4–S6, ESI†). Interestingly, all cells incubated with cationic 1 showed a significant shift towards less negative values when compared to controls or cells treated with either neutral or anionic DBCO probes 2 and 3. These differences can be ascribed to the partial neutralization of the cell surface negative charges upon “clicking” the cationic moiety, which is not observed when neutral label 2 is conjugated. In the case of the anionic label 3, a change towards more negative values is mostly observed for cancer HeLa and MDA-MB231 cells.

**Fig. 2:**
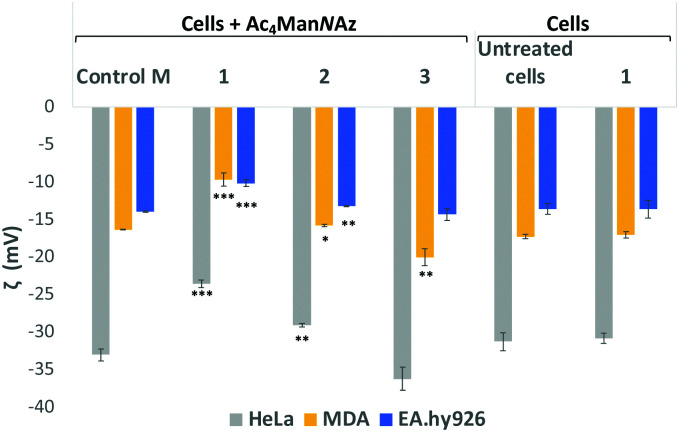
Zeta potential measurements of Ac_4_Man*N*Az treated cells which were incubated with DBCO-probes 1–3 for 1 hour. Cells were detached from the surface using TrypLE express solution and treated equally prior to zeta potential maeasurements (ESI† for details). Values are compared to controls Ac_4_Man*N*Az treated cells (Control M), untreated cells and cells just exposed to DBCO-1 for 1 h. Dotted lines are placed for the graphical comparison of the results with the controls. ns: *p* > 0.05, *: *p* < 0.05, **: *p* < 0.01, ***: *p* < 0.001.

In order to further confirmed that the changes observed on the zeta potential are linked to modification of cell surface sialic acids with our DBCO-probes, HeLa, MDA and EA·hy926 cells that had been incubated with 25 μM Ac_4_Man*N*Az, were then treated with neuraminidase from *Arthrobacter ureafaciens* to cleave all sialic acid residues prior to the 2nd labelling step with DBCO 1 (Fig. S8, ESI†). A general shift towards less negative zeta potential values when compared to cells treated with Ac_4_Man*N*Az alone (Control M) was observed with the change towards less negative values being more significant for EA·hy926. Moreover, the zeta potential values after neuraminidase treatment were similar to those obtained for Ac_4_Man*N*Az/DBCO-1 treated HeLa and MDA cells (not subjected to sialic acid cleaving). These results are consistent with the loss of sialic acids on the cell surface and also suggest that cationic labels such as 1 are able to neutralise the negative cell surface charges with a similar efficiency to the neuraminidase treatment, while maintaining sialic acid moieties on the glycans.

Another aspect we wanted to evaluate was whether modifying the cell surface charges could affect drug susceptibility on labelled cells. To that end, Alamar Blue and Calcein AM were used to quantify metabolic activity and the number of live cells, respectively. HeLa, MDA and EA·hy926 cells were incubated with Ac_4_Man*N*Az followed by labelling with DBCO-probes 1–3 were then exposed to a range of concentrations (0–1.4 mM) of doxorubicin, a model anticancer drug used to treat a wide range of cancers. In general, no significant differences in cell viability were observed between DBCO 1–3 labelled cells and controls (*e.g.* untreated cells or Ac_4_Man*N*Az treated cells) across all cell lines (see for details, Fig. S9–S14, ESI†), suggesting that the changes in surface charges induced by our labels do not have an effect on the cell drug response.

Migration study: Cell migration, invasion and adhesion are key steps in the development of cancer.^36^ Hypothesize that labelling with our probes could affect cell migration, particularly in cells where there is an overexpression of surface sialic acids. To that end, the wound healing assay is a standard *in vitro* technique for probing collective cell migration in two dimensions.^37^ In this assay, a cell-free area is created in a confluent monolayer by physical exclusion from and area through mechanical, thermal or chemical damage. A standard cell scratch assay used for cell migration and wound healing studies in culture multi-well microplates was used.^38^ A cell monolayer was established in 6 well plates and a scratch performed in each well to generate a cell-free area, cell migration from both sides of the wound was then monitored over 9 h and wound healing parameters including wound area, wound closure percentage and rate of closure were monitored over time for wounded cell monolayers that had been treated with the two step protocol (Ac_4_Man*N*Az/DBCO-probes 1–3) and compared to untreated cells (Control) in cell culture medium (see ESI† Section 2.7 for details). Time-lapse images were taken every 90 minutes (Fig. 3A–D for HeLa, and Fig. S15 and S16 in ESI† for MDA and EA·hy926 respectively) and analysed with ImageJ. Wound area analysis showed that wound closure is impeded in cells that were “clicked” with 25 μM of cationic DBCO-1 when compared to controls and showed a two-fold reduction on the rate of wound closure for HeLa cells (58% after 9 h compared to control), whilst HeLa cells labelled with 2 and 3 showed a similar behaviour to the control exhibiting around 60% wound closure after 7.5 h (Fig. 3E). MDA cells also showed a wound closure rate reduction albeit more moderate of around 10% (after 7.5 h when labelled with 1 (Fig. S17, ESI†). Similarly, for EA·hy926 cells no significant differences were observed between the different labelled cells and control. In order to confirm that the changes in cell migration are a consequence of the sialic acid charge neutralization on the cell surface, native HeLa cells were subjected to neuraminidase treatment prior to the double labelling protocol with DBCO-1. As expected, cells which had cell surface sialic acids removed showed a similar rate of wound closure to controls, which is independent from the DBCO labelling, further supporting that both cell surface charges and the type of terminal glycan play a pivotal role in modulating cell motility factors (Fig. S18, ESI†).

**Fig. 3:**
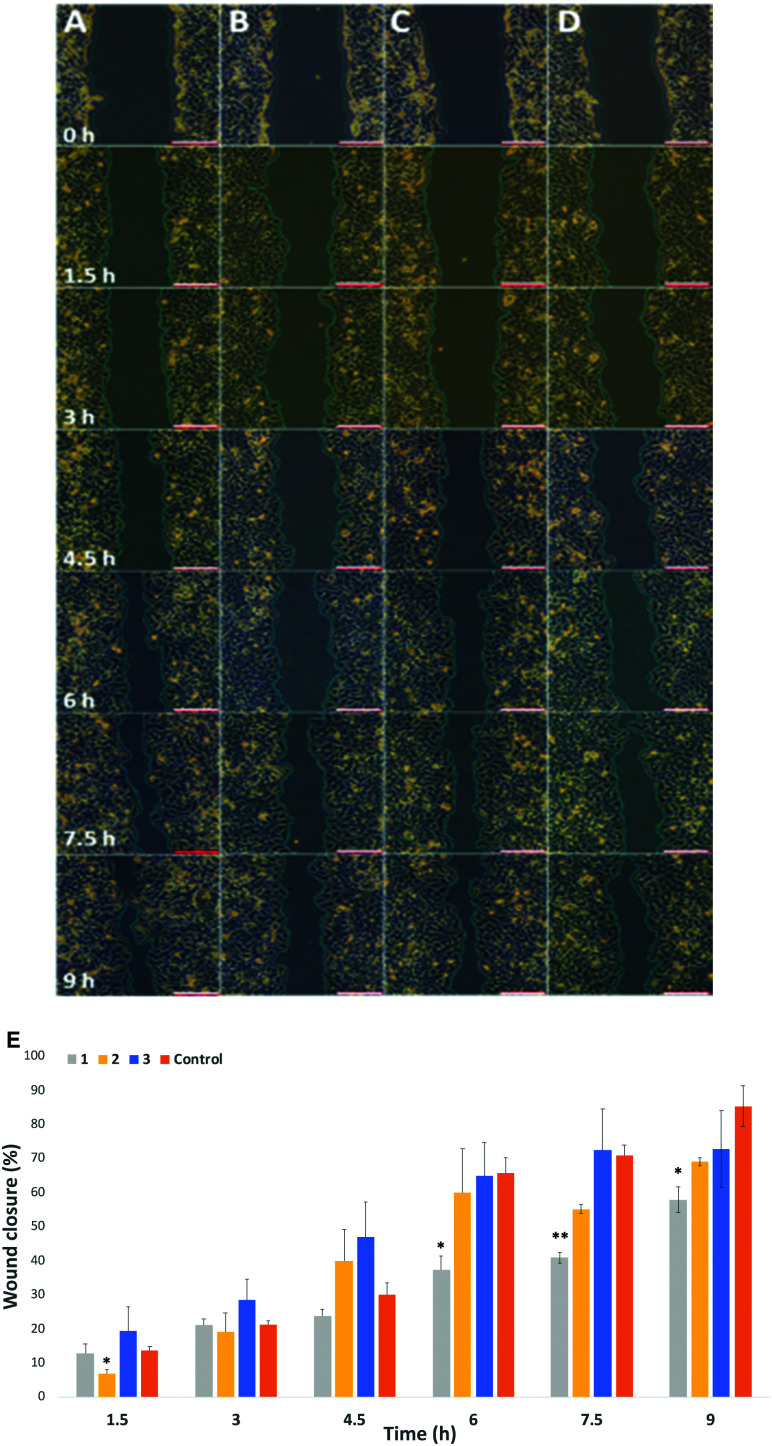
Representative scratch assay test for HeLa cells: (A) untreated cells (Control); (B) DBCO 1 functionalized cells; (C) DBCO 2 functionalized cells and (D) DBCO 3 functionalized cells. (E) Wound closure rate of HeLa cells, White scale bars = 200 μM distance. Snapshots are taken at the timeframe indicated on column A. ns: *p* > 0.05, *: *p* < 0.05, **:*p* < 0.05.

In conclusion, we have developed a new strategy to reengineer the cell surface charges of live cells which combines metabolic oligosaccharide engineering with a new class of DBCO-clickable tags. The new labels are easily accessible in one step and good yields from commercial starting materials and the glycan derivatization is efficient (>70% of azide containing surface glycans) within 1 h. More importantly, using this strategy we show that overall cell surface charges can be modulated and we demonstrate that neutralising the negative charges due to sialic glycan overexpression in cancer cells, while leaving the glycan moiety, leads to a reduction in cell migration, while drug susceptibility remains unchanged. These results highlight the importance of cell surface charges that are linked to glycan changes associated to disease progression, and how modulation of those might help control cell adhesion and migration. It is our hope that the probes we have developed herein will help pave the way for the development of novel therapeutics.

MCG thanks the European Research Council (ERC-COG: 648239).

## Conflicts of interest

There are no conflicts to declare.

## Supplementary Material

CC-058-D2CC00402J-s001
