# Supplementary material for: Reengineering of cancer cell surface charges can modulate cell migration
Source: Chem Commun (Camb). 2022 Apr 6;58(36):5522–5. doi: 10.1039/d2cc00402j (PMC9063860; doi:10.1039/d2cc00402j)
Supplement: CC-058-D2CC00402J-s001 [file CC-058-D2CC00402J-s001.pdf]

Electronic Supplementary Material (ESI) for ChemComm.
This journal is © The Royal Society of Chemistry 2022

Supporting Information

Reengineering of cancer cell surface charges via metabolic oligosaccharide engineering can tune cell adhesion

Mattia Ghirardello, Radhe Shyam, and M. Carmen Galan*

School of Chemistry, University of Bristol, Bristol, United Kingdom.
E-mail: m.c.galan@bristol.ac.uk

TABLE OF CONTENTS

1. Chemical synthesis of DBCO tags 1-3. ..... S2
   1.1. Materials and methods S2
   1.2. Positively charged DBCO Tag (**1**) S2
   1.3. Neutrally charged DBCO Tag (**2**) S4
   1.4. Negatively charged DBCO Tag (**3**) S6
2. Biological studies S7
   2.1. Cell culture protocols S7
   2.2. Cell metabolic activity S7
   2.3. Determination of DBCO tag labelling efficiency with Cy5 fluorescence analysis S8
   2.4. Zeta potential analysis S9
   2.5. Zeta potential of neuraminidase-treated cells S9
   2.6. Drug susceptibility study S10
   2.7. Cell migration analysis S13
3. Supplementary references S17

1. Chemical synthesis of DBCO tags 1-3.

1.1. Materials and methods.

Chemicals were purchased and used without further purification. Dry solvents were obtained by distillation using standard procedures or by passage through a column of anhydrous alumina using equipment from Anhydrous Engineering (University of Bristol) based on the Grubbs' design. Reactions requiring anhydrous conditions were performed under nitrogen; glassware and needles were either flame dried immediately prior to use or placed in an oven (150 °C) for at least 2 hours and allowed to cool either in a desiccator or under reduced pressure; liquid reagents, solutions or solvents were added via syringe through rubber septa; solid reagents were added via Schlenk type adapters. Teflon rings were used between the joints of the condensers and round bottom flasks. Reactions were monitored by TLC on Kieselgel 60 F254 (Merck). Detection was by examination under UV light (254 nm) and by staining with cerium ammonium sulphate (CAM) dip. Flash column chromatography was performed using silica gel [Merck, 230−400 mesh (40−63 µm)]. Extracts were concentrated in vacuo using both a Buchi rotary evaporator (bath temperatures up to 40 °C) at a pressure of either 15 mmHg (diaphragm pump) or 0.1 mmHg (oil pump), as appropriate, and a high vacuum line at room temperature. Preparative HPLC was performed on a Grace Discovery Sciences Reveleris Prep System with a Phenomenex Luna a Luna PREP C18 column (10 µm, 250 x 21.2 mm) using the gradient stated in the experimental description of each compound. ^{1}H NMR and ^{13}C NMR spectra were recorded on Bruker AV 400 MHz or AV 500 MHz spectrometers, using the residual solvent peaks as internal reference at 298 K. Chemical shifts are quoted in parts per million from residual solvent peak ($CDCl_3$: ^{1}H: 7.26 ppm and ^{13}C: 77.16 ppm) and coupling constants (J) given in Hertz. Multiplicities are abbreviated as: b (broad), s (singlet), d (doublet), t (triplet), q (quartet), m (multiplet) or combinations thereof. Electrospray ionisation (ESI) mass spectra were recorded on a Micromass LCT mass spectrometer or aVG Quattro mass spectrometer.
The NMR assignment follows the numeration reported in the exemplificative figure below.

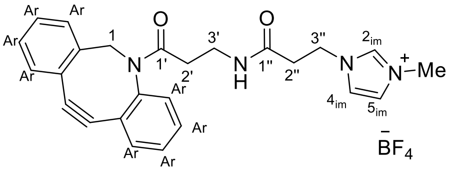

1.2. Positively charged DBCO Tag (1).

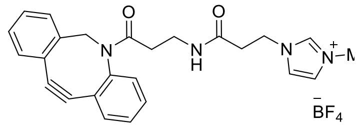

To a stirred suspension of acid **5**[1] (114 mg, 0.47 mmol) in dry CH_3CN (5 mL), EDC·HCl (134 mg, 0.66 mmol) and NHS (76 mg, 0.66 mmol) were added as solid at room temperature and the solution was stirred under inert atmosphere for 16 h at room temperature. A solution of **4** (65 mg, 0.24 mmol) in dry CH_3CN (0.5 mL) was added to the reaction mixture, the flask containing the solution of **4** was rinsed with further dry CH_3CN (0.5 mL) and added to the reaction mixture. The solution was stirred for 5 h at room temperature under inert atmosphere and then concentrated under reduced pressure. The residue was purified by RP-HPLC using a linear gradient of 5-95 % acetonitrile/water over 20 min at a flow rate of 14 mL/min furnishing **1** (55 mg, 46 % yield) as a transparent syrup.

^{1}H NMR (500 MHz, Methanol-d_4) δ 8.82 (s, 1H, H2$_{im}$), 7.68 – 7.64 (m, 1H, Ar), 7.55 – 7.43 (m, 6H, Ar), 7.34 (dtd, *J* = 18.0, 7.4, 1.5 Hz, 2H, Ar), 7.26 (dd, *J* = 7.3, 1.6 Hz, 1H, Ar), 5.14 (d, *J* = 14.1 Hz, 1H, H1$_a$), 4.34 (qdd, *J* = 14.0, 6.8, 5.5 Hz, 2H, H3$^{II}_a$, H3$^{II}_b$), 3.90 (s, 3H, CH_3), 3.71 (d, *J* = 14.0 Hz, 1H, H1$_b$), 3.22 –

3.12 (m, 2H, H3'$_a$, H3'$_b$), 2.62 – 2.58 (m, 2H, H2''$_a$, H2''$_b$), 2.46 (dt, *J* = 15.9, 6.2 Hz, 1H, H2'$_a$), 2.04 (dt, *J* = 16.0, 7.2 Hz, 1H, H2'$_b$).

^{13}C NMR (126 MHz, Methanol-*d*$_4$) δ 172.8, 171.2, 152.6, 149.6, 133.4, 130.5, 130.0, 129.8, 129.3, 129.0, 128.2, 126.5, 124.7, 124.3, 123.7, 123.7, 115.6, 108.8, 56.6, 46.7, 36.7, 36.5, 36.2, 35.3.

HRMS (ESI) m/z: Calcd for $C_{25}H_{25}N_4O_2^+$ (M)$^+$ 413.1972, found 413.1957.

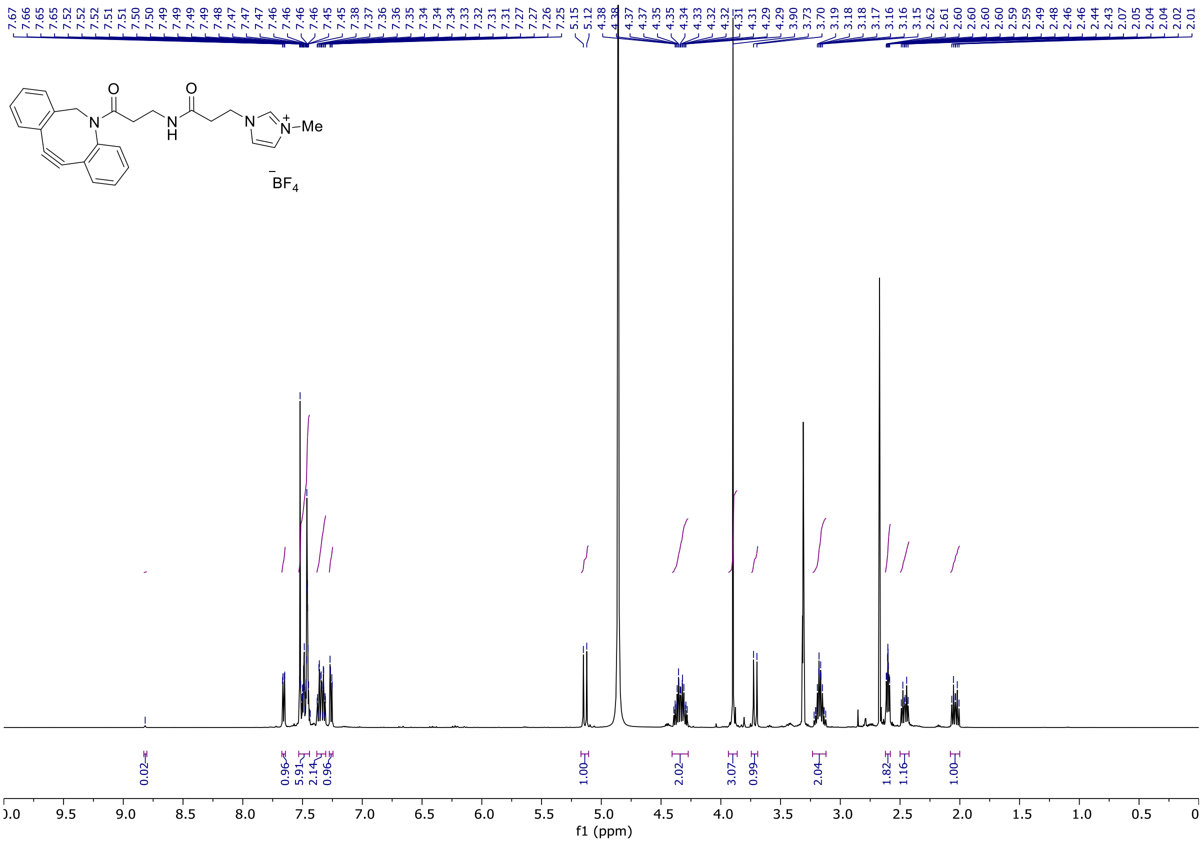

Figure S1. ^{1}H NMR spectrum of **1** (500 MHz, Methanol-*d*$_4$).

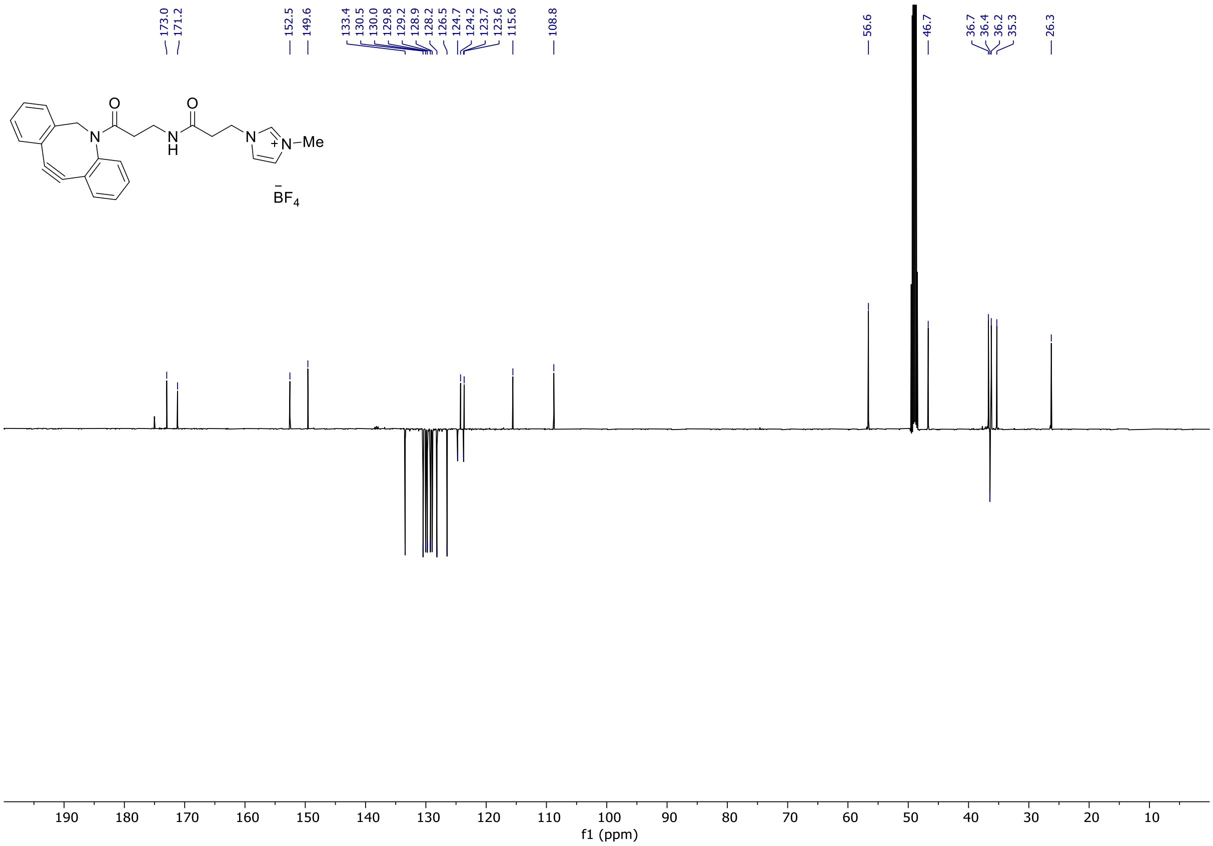

Figure S2. ^{13}C APT NMR spectrum of **1** (126 MHz, Methanol-d_4).

1.3. Neutrally charged DBCO Tag (2).

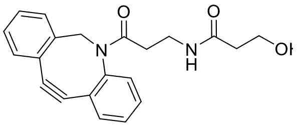

To a stirred solution of amine **4** (61 mg, 0.22 mmol) in toluene (1 mL), β-propiolactone (159 mg, 2.2 mmol) was added, and the mixture was stirred for 24 h at room temperature. The solution was concentrated under reduced pressure and the residue was purified by column chromatography on silica gel (EtOAc/MeOH 1:0 to 93:7, v/v) furnishing 2 (46 mg, 60 % yield) as a transparent film.

^{1}H NMR (400 MHz, $CDCl_3$) δ 7.66 (dd, *J* = 7.7, 1.4 Hz, 1H, Ar), 7.42 – 7.28 (m, 6H, Ar), 7.26 (dd, *J* = 7.5, 1.6 Hz, 1H, Ar), 6.33 (d, *J* = 6.2 Hz, 1H, NH), 5.12 (d, *J* = 13.9 Hz, 1H, $H1_a$), 3.79 – 3.65 (m, 3H, $H1_b$, $H3^{II}_a$, $H3^{II}_b$), 3.35 (dddd, *J* = 13.8, 7.6, 6.3, 4.0 Hz, 1H, $H3^{I}_a$), 3.21 (dddd, *J* = 13.5, 7.4, 5.9, 3.9 Hz, 1H, $H3^{I}_b$), 2.44 (ddd, *J* = 16.5, 7.4, 4.0 Hz, 1H, $H2^{I}_a$), 2.23 (t, *J* = 5.5 Hz, 2H, $H2^{II}_a$, $H2^{II}_b$), 1.98 (ddd, *J* = 16.5, 7.6, 3.8 Hz, 1H, $H2^{I}_b$).
^{13}C NMR (101 MHz, $CDCl_3$) δ 172.4, 172.3, 151.2, 148.1, 132.3, 129.1, 128.8, 128.6, 128.4, 128.0, 127.4, 125.7, 123.1, 122.6, 114.8, 107.9, 59.0, 55.6, 37.9, 35.3, 34.7.
HRMS (ESI) m/z: Calcd for $C_{21}H_{20}N_2NaO_3$ $(M+Na)^+$ 371.1366, found 371.1380.

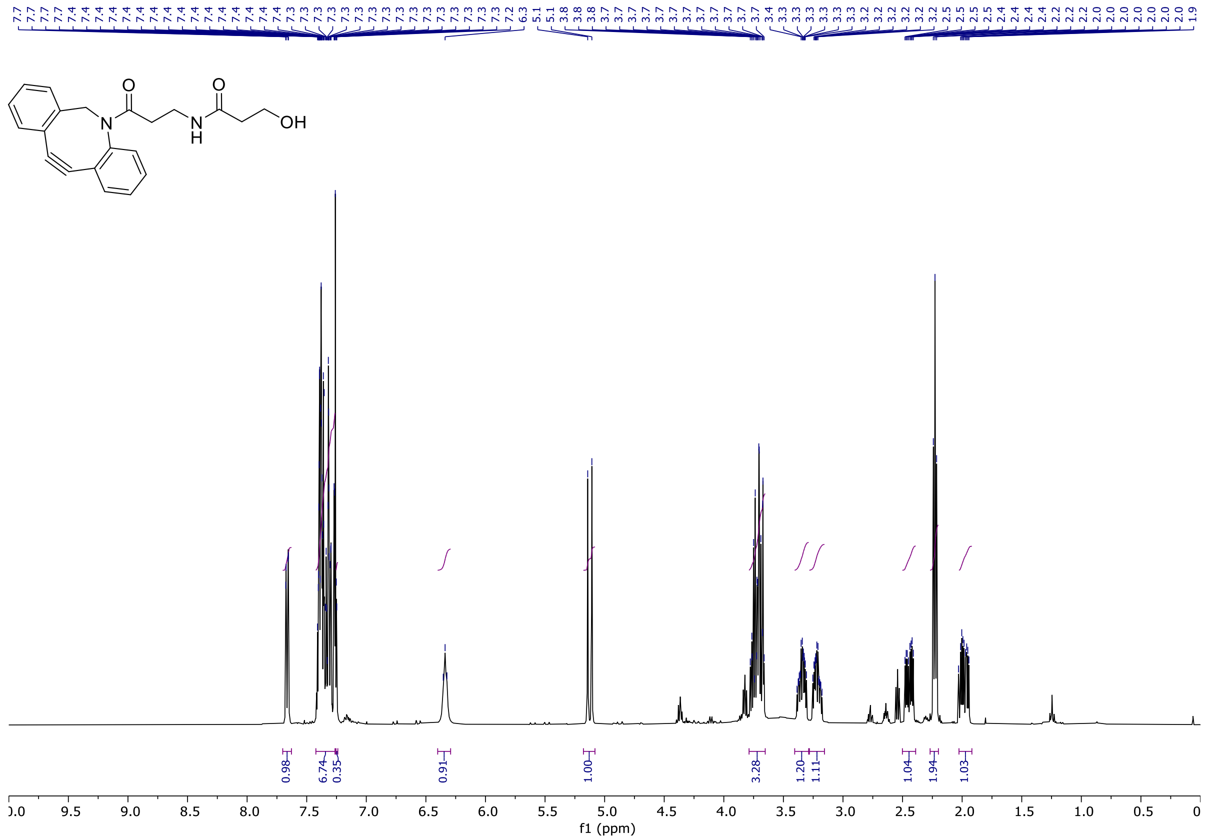

Figure S3. ^{1}H NMR spectrum of **2** (400 MHz, $CDCl_3$).

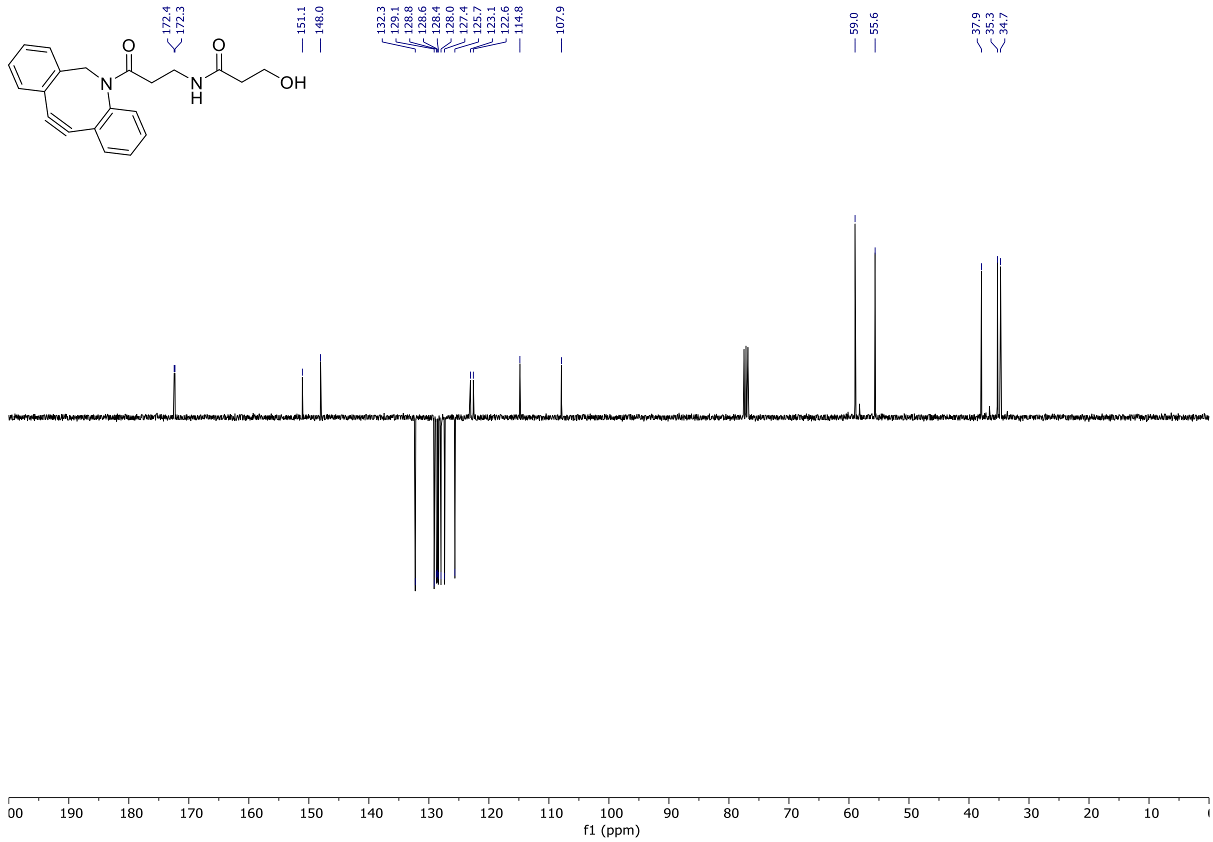

Figure S4. ^{13}C APT NMR spectrum of **2**(101 MHz, $CDCl_3$).

1.4. Negatively charged DBCO Tag (3).

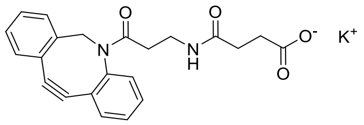

To a stirred solution of amine **4** (145 mg, 0.53 mmol) in dry CH_2Cl_2 (10 mL), succinic anhydride (105 mg, 1.05 mmol) was added, and the mixture was stirred under inert atmosphere for 16 h at room temperature. The solution was concentrated under reduced pressure, the residue was redissolved in MeOH (2 mL) and treated with K_2CO_3 (291 mg, 2.1 mmol) added as a H_2O solution (2 mL). The mixture was stirred for 2 h at room temperature and then concentrated under reduced pressure. The residue was purified by RP-HPLC using a linear gradient of 5-95 % acetonitrile/water over 20 min at a flow rate of 14 mL/min furnishing **3** (116 mg, 53 % yield) as a transparent syrup.

^{1}H NMR (400 MHz, Methanol-d_4) δ 7.65 (dd, *J* = 7.3, 1.6 Hz, 1H, Ar), 7.51 – 7.41 (m, 4H, Ar), 7.39 – 7.30 (m, 2H, Ar), 7.28 – 7.24 (m, 1H, Ar), 5.13 (d, *J* = 14.0 Hz, 1H, $H1_a$), 3.69 (d, *J* = 14.0 Hz, 1H, $H1_b$), 3.25 (ddd, *J* = 13.6, 7.3, 6.3 Hz, 1H, $H3^{I}_{a}$), 3.13 (dt, *J* = 13.6, 7.0 Hz, 1H, $H3^{I}_{b}$), 2.53 – 2.42 (m, 1H, $H2^{I}_{a}$), 2.36 – 2.26 (m, 4H, $H2^{II}_{a}$, $H2^{II}_{b}$, $H3^{II}_{a}$, $H3^{II}_{b}$), 2.02 (dt, *J* = 15.9, 7.1 Hz, 1H, $H2^{I}_{b}$).
^{13}C NMR (101 MHz, Methanol-d_4) δ 180.8, 175.7, 173.3, 152.6, 149.4, 133.4, 130.5, 130.0, 129.7, 129.2, 128.9, 128.1, 126.6, 124.3, 123.7, 115.7, 108.8, 56.6, 36.6, 35.5, 34.5, 33.9.
HRMS (ESI) m/z: Calcd for $C_{22}H_{19}N_2O_4^-$ $(M)^-$ 375.1345, found 375.1344.

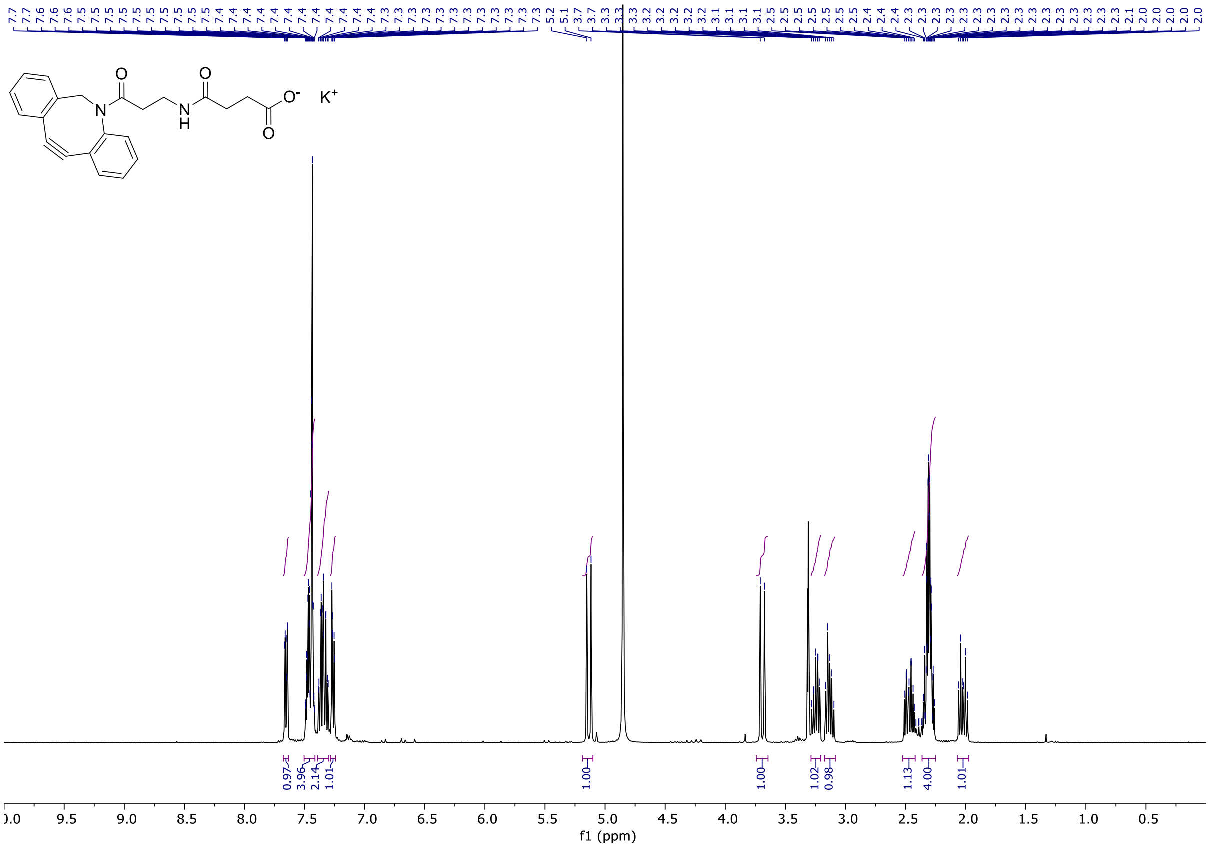

Figure S5. ^{1}H NMR spectrum of **3** (400 MHz, Methanol-d_4).

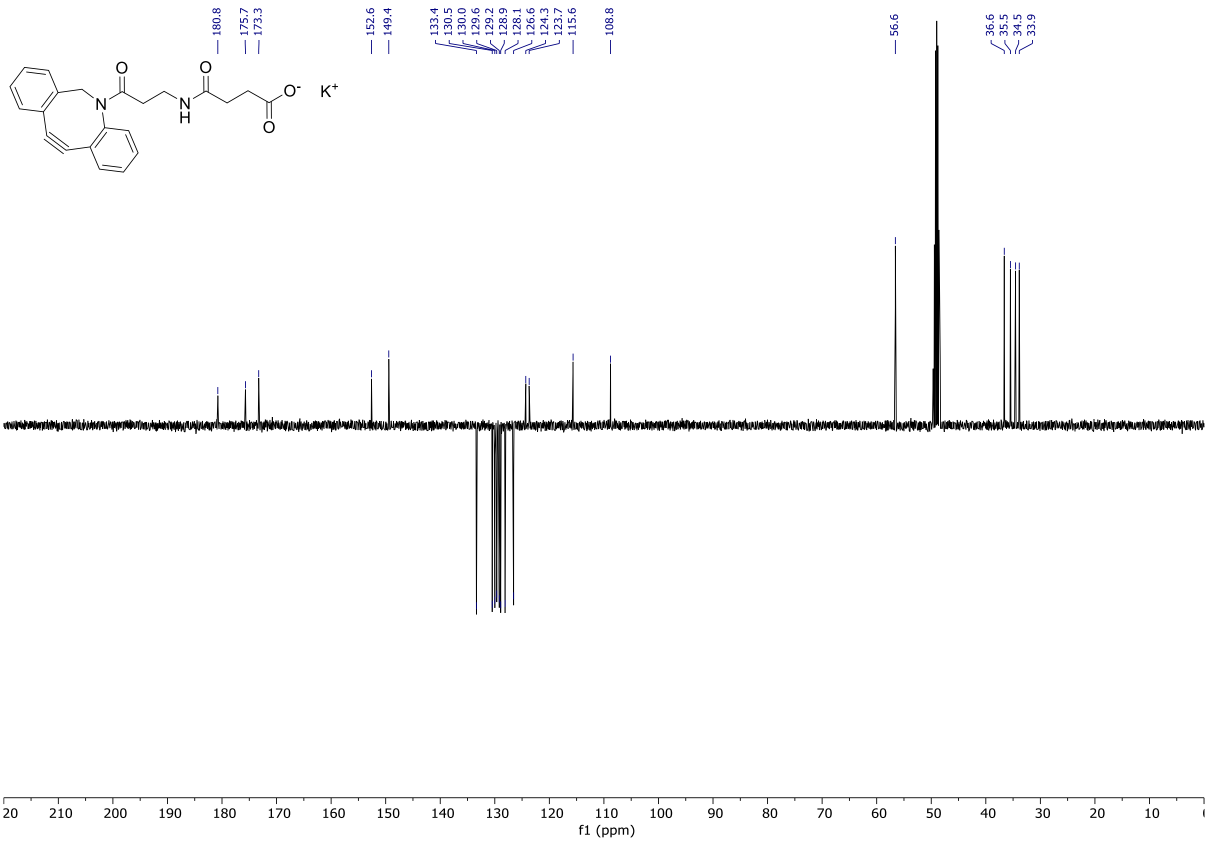

Figure S6. ^{13}C APT NMR spectrum of **3** (101 MHz, Methanol-d_4).

2. Biological studies.

2.1. Cell culture protocols.

HeLa – Human cervical carcinoma cell line ATCC® CCL-2™ (HeLa) was grown in Dulbecco's Minimal Essential Medium (DMEM) – high glucose (4.5g/L D-glucose).

EA.hy926 – hybrid somatic cell line ATCC® CRL-2922™ was grown in Dulbecco's Minimal Essential Medium (DMEM) – high glucose (4.5g/L D-glucose).

MDA-MB-231 Human adenocarcinoma cell line ATCC® CRM-HTB-26™ was grown in Dulbecco's Minimal Essential Medium (DMEM) – high glucose (4.5g/L D-glucose).

All growth media were supplemented with antibiotic-antimycotic (Anti-Anti) and 10% fetal bovine serum (FBS); all cell culture media were purchased from Invitrogen, Life technologies. Confluent cultures were detached from the surface using trypsin (TrypLE Express, Invitrogen) and plated at $2*10^4$ cells per/well in 6 well plates for cell scratch assay.

The gain for the fluorescent readings was previously adjusted for HDF and HeLa plates, respectively, based on the well with the highest fluorescent value of each plate. Fluorescent readings for the standard curves were acquired with the CLARIOstar® microplate reader (BMG LABTECH, UK).

2.2. Cell metabolic activity.

Changes in cell metabolism were assessed using AlamarBlue (AB, Life Technologies), a cytosolic substrate for reductive metabolism (resazurin to resorufin) whose fluorescence spectrum changes on reduction by cytosolic enzymes. Cells were incubated with DBCO-probes **1-3** (1 h) in medium with reduced FBS (5%). The plates were washed with PBS, and AB (5 % solution), was added in medium

without FBS. After 1.5 hour incubation, the fluorescence of the dye was read using a plate reader (BMG Labtech CLARIOstar) (AB λex = 545 nm λem = 590 nm). Results are expressed as relative metabolic activity (%) compared to untreated cell controls (set as 100 %). Experiments are repeated at least as triplicates the histogram bars represents the average of the relative metabolic activity and error bars represents the standard error of the mean.

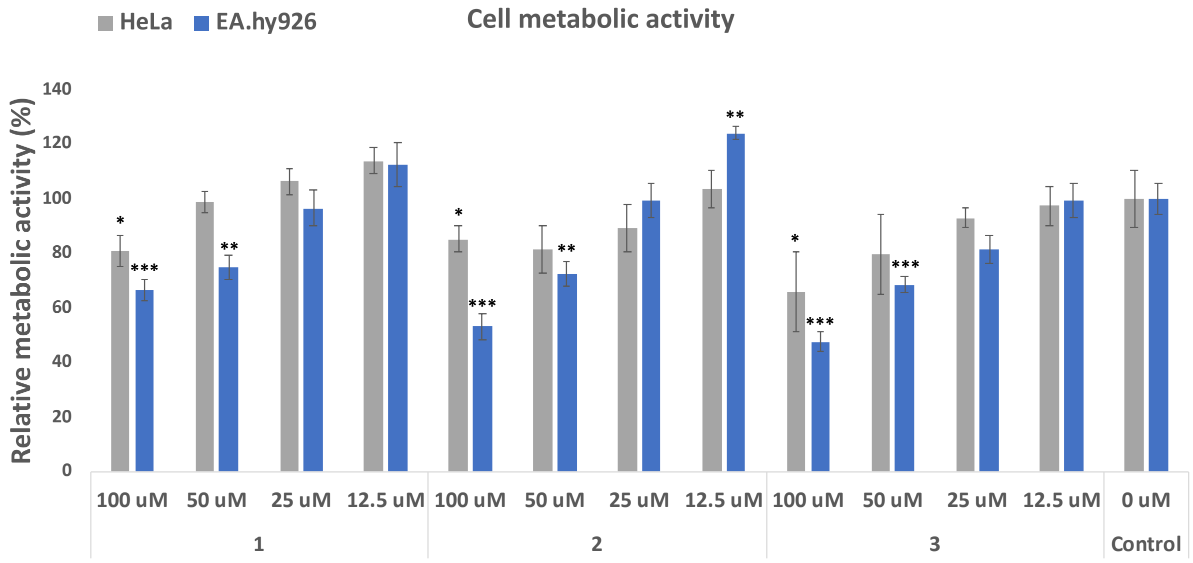

Figure S7. Cell metabolic activity of EA.hy926 and HeLa cell lines incubated with variable amounts of DBCO tags **1-3**. Statistical significance versus Control group using unpaired Student's t-test:*p<0.05, **p<0.01, ***p<0.001

2.3. Determination of DBCO tag labelling efficiency with Cy5 fluorescence analysis.

Hela and MDA cells were incubated in just media or media containing 25 uM Ac_4Man*N*Az. After 3 days, the cells were washed with PBS (2X). The cells were then incubated for 1h (unless otherwise noted) at 37 °C with the desired reagent (no reagent, cationic DBCO-**1**, neutral DBCO-**2**, or anionic DBCO-**3**).

Table S1. Fluorescent determination of HeLa cell labelling.

**HeLa**	Cells + Ac_4ManNAz + Cy5				Cells + Cy5			
	**Control M**	**1**	**2**	**3**	**Control**	**1**	**2**	**3**
Average	26210.50	7314.00	7313.75	7410.50	0.00	1801.50	1509.00	2293.50
Standard Error of Mean	675.04	941.58	890.26	346.12	1464.18	2092.91	1631.37	984.72
Relative fluorescence labelling (%)	100.00	27.90	27.90	28.27	0.00	6.87	5.76	8.75

Table S2. Fluorescent determination of MDA cell labelling.

**MDA**	Cells + Ac_4ManNAz + Cy5				Cells + Cy5			
	**Control M**	**1**	**2**	**3**	**Control**	**1**	**2**	**3**
Average	47651.75	12410.50	12894.50	15205.25	0.00	3631.50	2258.25	5213.00
Standard Error of Mean	1173.09	3793.84	1630.99	1649.28	7296.03	2184.41	3399.03	3332.36
Relative fluorescence labelling (%)	100.00	26.04	27.06	31.91	0.00	7.62	4.74	10.94

Table S3. Fluorescent determination of EA-hy926 cell labelling.

**EA.hy926**	Cells + Ac_4ManNAz + Cy5	Cells + Cy5

	Control M	1	2	3	Control	1	2	3
Average	59211.88	7322.38	8775.75	10706.88	0.00	9340.00	5206.38	5605.63
Standard Error of Mean	5780.88	647.95	1849.30	2580.02	372.89	5345.66	348.03	639.17
Relative fluorescence labelling (%)	100.00	12.37	14.82	18.08	0.00	15.77	8.79	9.47

2.4. Zeta potential analysis.

Zeta potential analysis were carried out using a Malvern Instruments Nano-Z Zen 2600 in 0.1M PBS. Cells were either untreated or treated with 25 µM $Ac_4ManNAz$ for 3 days. Afterwards the cells were treated with probes (no reagent, 1, 2, or 3) for 2 hours before measuring the Zeta potential. The results are given in the tables below.

Table S4. Zeta potential for HeLa cells treated with DBCO Tags and with neuraminidase.

HeLa	Cells + Ac4ManNAz				Untreated cells		Neuraminidase	
	Control M	**1**	**2**	**3**	Blank	Blank + **1**	**1** + neuraminidase	Neuraminidase +**1**
Repeat 1	-32.9	-23.7	-29.4	-36.3	-29.6	-31.4	-23.6	-24.6
Repeat 2	-34	-22.9	-28.9	-38	-32.1	-31.2	-22.4	-24.5
Repeat 3	-32.1	-24.1	-28.9	-34.3	-32.1	-29.9	-22.1	-23.7

Table S5. Zeta potential for MDA cells treated with DBCO Tags and with neuraminidase.

MDA	Cells + Ac4ManNAz				Untreated cells		Neuraminidase	
	Control M	**1**	**2**	**3**	Blank	Blank + **1**	**1** + neuraminidase	Neuraminidase +**1**
Repeat 1	-16.4	-8.71	-16	-21.6	-17.6	-17.6	-10.5	-9.37
Repeat 2	-16.3	-10.8	-15.7	-19.5	-17.3	-16.7	-10.4	-9.1
Repeat 3	-16.3	-9.47	-15.6	-19	-16.9	-16.7	-10.1	-10.7

Table S6. Zeta potential for EA-hy926 cells treated with DBCO Tags and with neuraminidase.

EA.hy926	Cells + Ac4ManNAz				Untreated cells		Neuraminidase	
	Control M	**1**	**2**	**3**	Blank	Blank + **1**	**1** + neuraminidase	Neuraminidase +**1**
Repeat 1	-14	-10.7	-13.3	-13.4	-12.6	-12.9	-5.11	-5.05
Repeat 2	-13.9	-10.2	-13.2	-15.3	-14.1	-12.7	-3.45	-3.85
Repeat 3	-13.8	-9.58	-13.1	-14.2	-14	-15.2	-3.87	-4.64

2.5. Zeta potential of neuraminidase-treated cells.

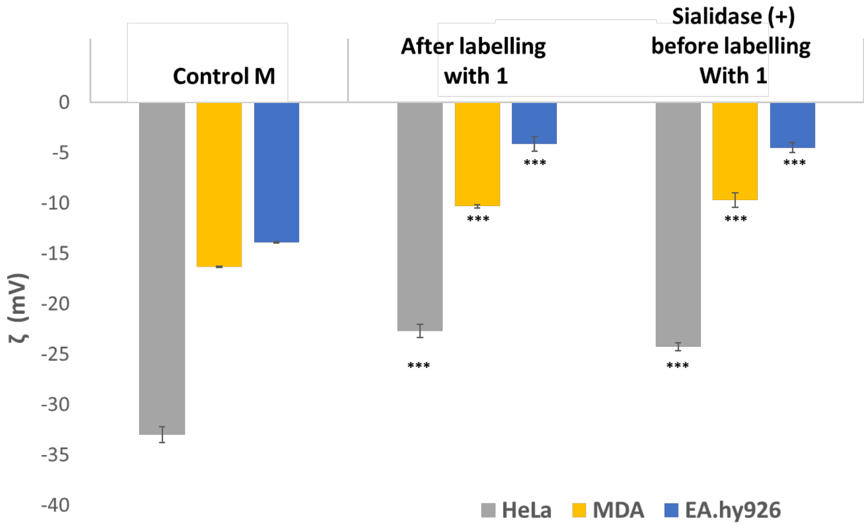

Figure S8. Zeta potential measurements for HeLa, MDA and EA.hy926 cells after 72 h incubation with $Ac_4ManNAz$ (25 mM) (control M); compared to cells incubated with $Ac_4ManNAz$ (25 mM) and labelled with **1** (After labeling with 1); and cells incubated with $Ac_4ManNAz$ (25 mM), treated with neuraminidase followed by treatment with **1** (Sialidase(+) before labeling with **1**). Statistical significance versus Control M group using unpaired Student's t-test: * p ≤ 0.05, ** p ≤ 0.01, *** p ≤ 0.001.

2.6. Drug susceptibility study.

The influence of DBCO-probes **1-3** and Doxorubicin on cell survival after exposure to the compounds was quantified by measuring calcein fluorescence. The fluorescence, retained within live cells only, results from activity of esterases on the (nonfluorescent) calcein AM (Molecular Probes). Changes in cell metabolism were assessed using AlamarBlue (AB, Life Technologies), a cytosolic substrate for reductive metabolism (resazurin to resorufin) whose fluorescence spectrum changes on reduction by cytosolic enzymes. MDA-MB-231, HeLa and EA.hy926 ($2x10^4$ cells) that were subjected to the labelling process with Ac_4ManNAz (48h) followed by DBCO-probes **1-3** (1.5h) were incubated with Doxorubicin (1.4 µM – 0.04 µM) for 48 hours. Each experiment was repeated at least twice, in medium with reduced FBS (5%), with each data point conducted in octuplicate. After 48h the plates were washed with PBS, and AB (5 % solution), calcein (3 µM) were added in medium without FBS. After 1.5 hour incubation, the fluorescence of both dyes was read using a plate reader (BMG Labtech CLARIOstar) (AB λex = 545 nm λem = 590 nm, calcein λex = 494 nm, λem = 517 nm). Results are expressed as percentages of 100% control, versus the doxorubicin concentration.

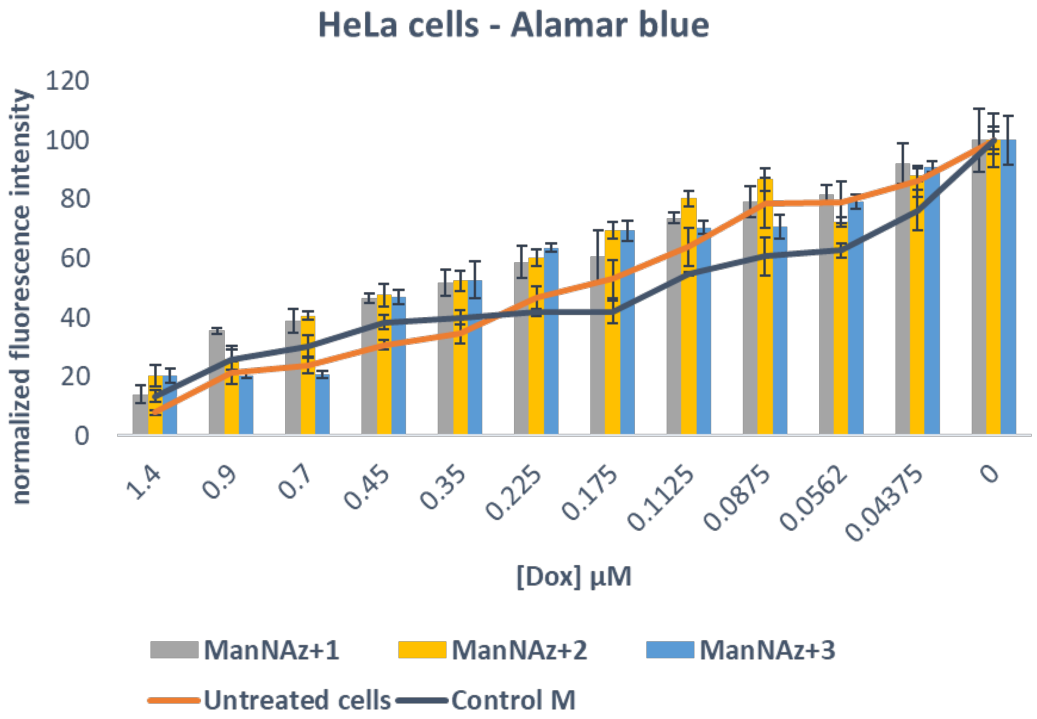

Figure S9. Effects on reductive metabolism (alamar Blue assay) for HeLa after 48h incubation with doxorubicin and compounds **1-3**.

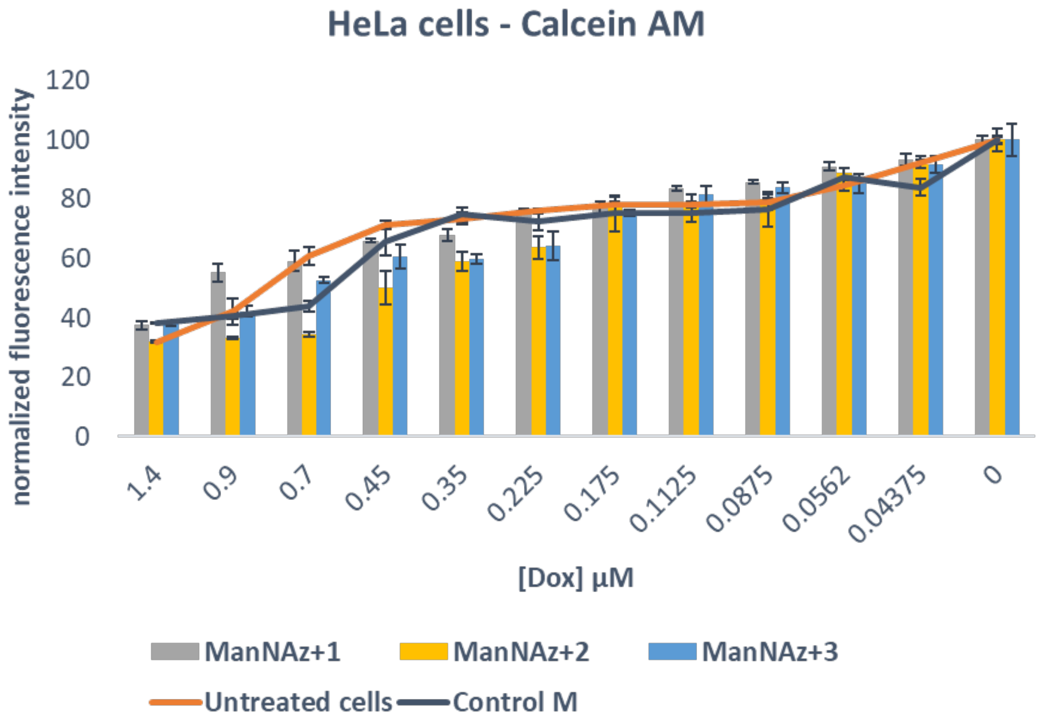

Figure S10. Calcein AM assay for HeLa with Doxorubicin and compounds **1-3.**

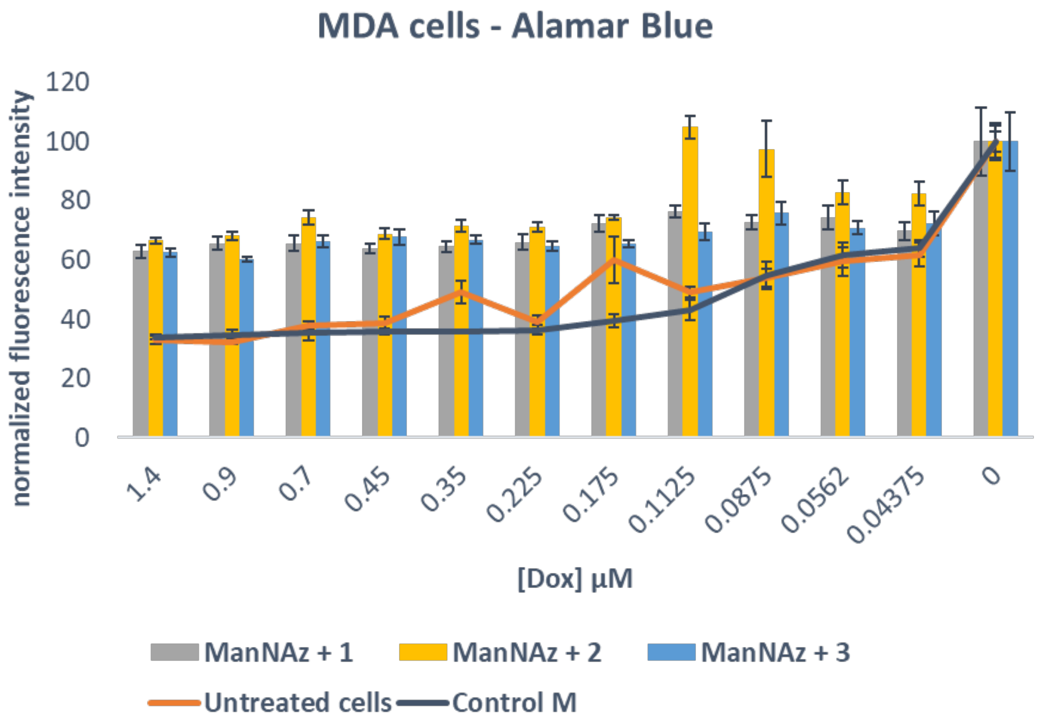

Figure S11. Effects on reductive metabolism (alamar Blue assay) for MDA after 48h incubation with doxorubicin and compounds **1-3**.

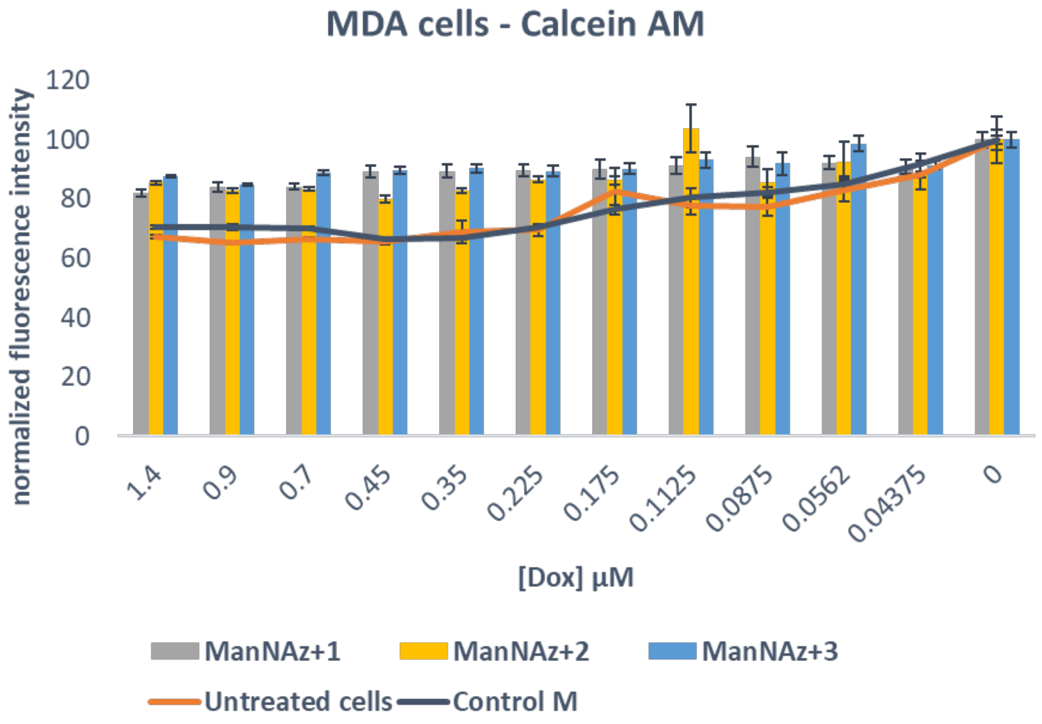

Figure S12. Calcein AM assay for MDA with Doxorubicin and **1-3**.

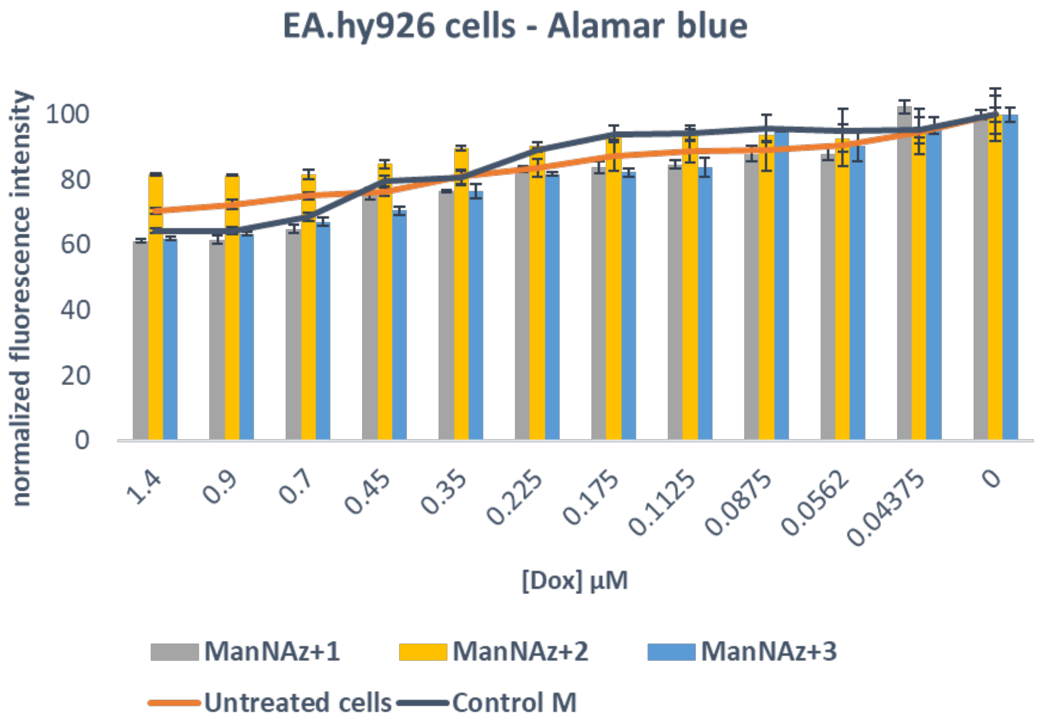

Figure S13. Effects on reductive metabolism (alamar Blue assay) for EA.hy926 after 48h incubation with doxorubicin and compounds **1-3**.

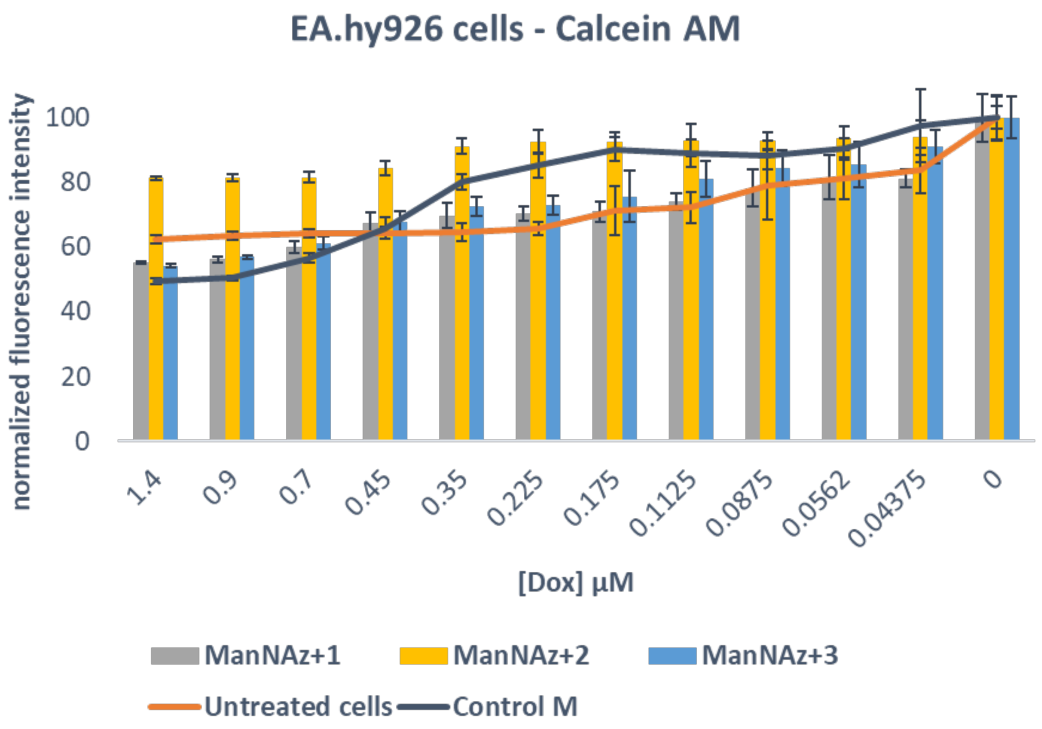

Figure S14. Calcein AM assay for EA.hy926 with Doxorubicin and compounds **1-3**.

2.7. Cell migration analysis.

A cell monolayer was established in a 6 well plates and a cell scratch was performed in each well to generate a cell-free area using a p200 micropipette tip, cell migration from both sides of the wound was then monitored over 6 h and wound healing parameters including wound area, and wound closure percentage were monitored over time for wounded cell monolayers cultivated which had been treated with the two step protocol (Ac_4Man*N*Az/DBCO-probes **1-3**) and compare to unlabelled cells (Control M) in cell culture medium. Time-lapse images were taken every 90 minutes and analysed with ImageJ.

After wounding, the wound closure was monitored manually using a DMIL fluorescence microscope (Leica). Samples were positioned on the microscope stage and the light intensity was adjusted. 4× magnification objective was selected for observation of the wounded area. Coarse and fine focus knobs were used to bring the specimen into optimal focus. The brightness of the image was adjusted, and pictures were taken in phase contrast mode.

Image analysis of cell-free areas using ImageJ software.

To ensure that pictures were always taken at the same position, the plates were marked using a fine marker and same position was focused, which allows resuming of previously used positions. Pictures were taken at 0, 1.5, 3, 4.5 and 6 hours. To analyse the images taken by microscopes, the pictures were exported in TIF format. After opening the images in Image J software, the unit for distance measurement was changed from pixel to micrometre (µm). This change of the scale was applied to all images prior to image analysis by drawing a free line over the scale bar and selecting the "Set Scale" key into desired unit.

The size of the gaps was measured at all time points using the MRI Wound healing tool (http://dev.mri.cnrs.fr/projects/imagej-macros/wiki/Wound_Healing_Tool) in ImageJ (https://imagej.nih.gov/ij/, version 2.0.0-rc-43/1.50e).

The wound areas were measured, and the average wound size was calculated at desired time points. Final wound area A(t) was subtracted from initial wound area A(t0) and this number was divided by the initial wound area as shown in Eq. (2).

$$Wound\ closure\ (\%) = (1 - \frac{A\ (t)}{A\ (t0)}) * 100 \qquad (2)$$

To quantify the wound size reproducibility and be able to compare the wound size reproducibility between conventional scratch assay and wound healing assay-on-chip, wound areas were measured from multiple wounds. From multiple measurements, the average wound size ($\bar{X}$) and standard deviation (SD) were calculated.

For unbiased automated image analysis of wound closure, the thresholding tool of ImageJ was used prior to analysis of wound closure and cell migration.

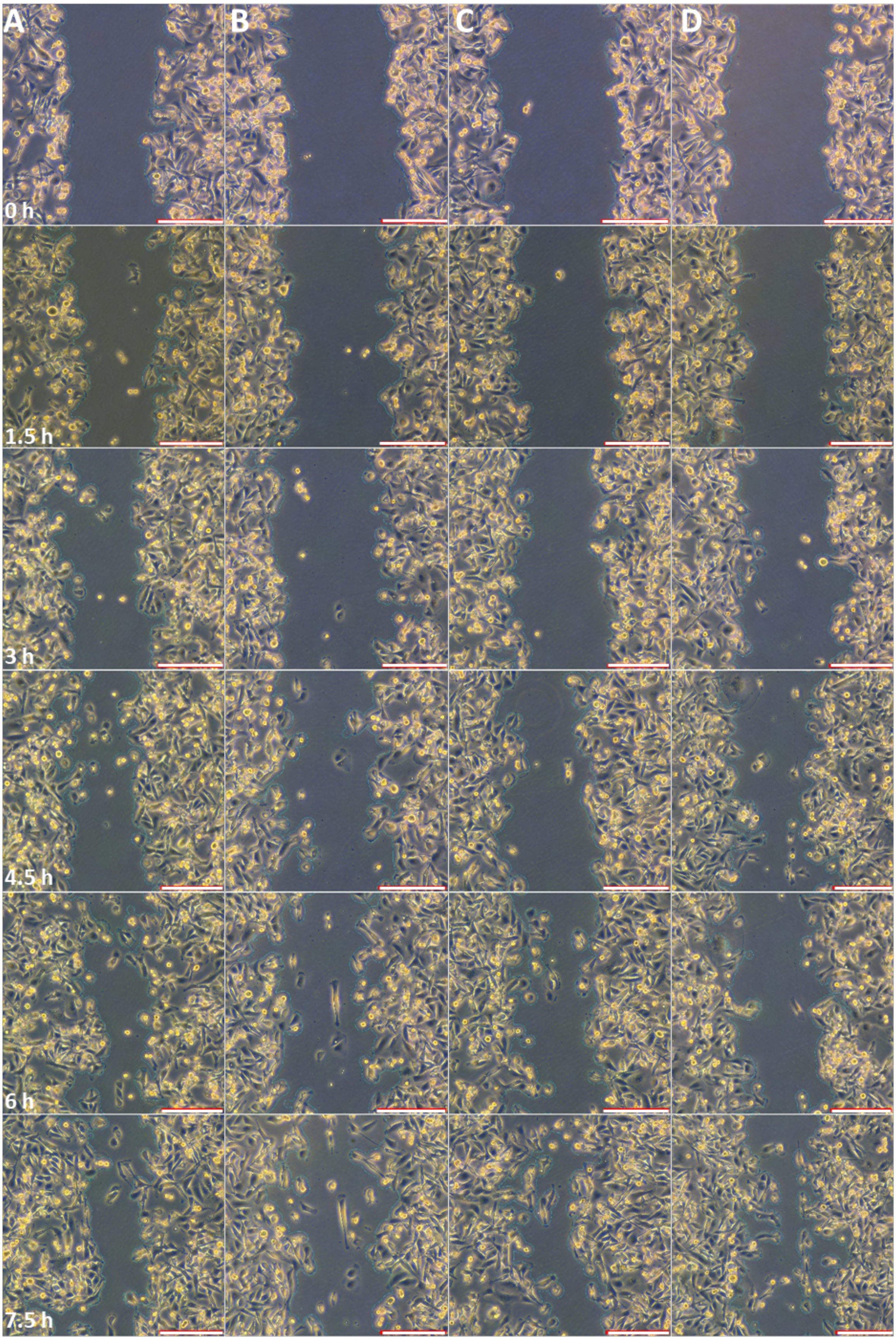

Figure S15. Scratch assay test on MDA cells: A) $Ac_4ManNAz$ treated cells (Control M); B) DBCO **1** functionalized cells; C) DBCO **2** functionalized cells and D) DBCO **3** functionalized cells. Snapshots are taken at the timeframe indicated on column A. White scale bars indicate a 200 µM distance.

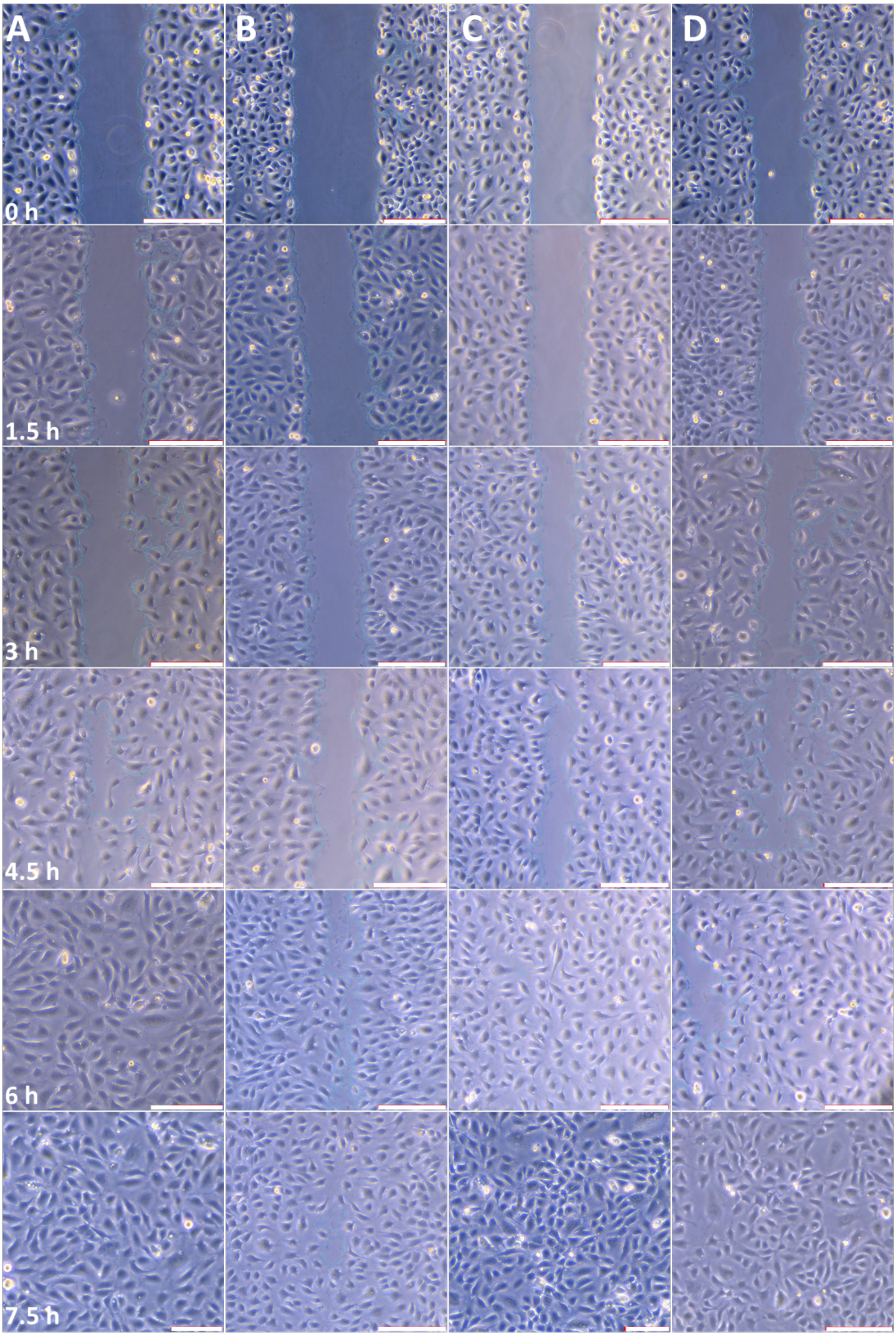

Figure S16. Scratch assay test on EA.hy926 cells: A) Ac_4Man*N*Az treated cells (Control M); B) DBCO **1** functionalized cells; C) DBCO **2** functionalized cells and D) DBCO **3** functionalized cells. Snapshots are taken at the timeframe indicated on column A. White scale bars indicate a 500 µM distance.

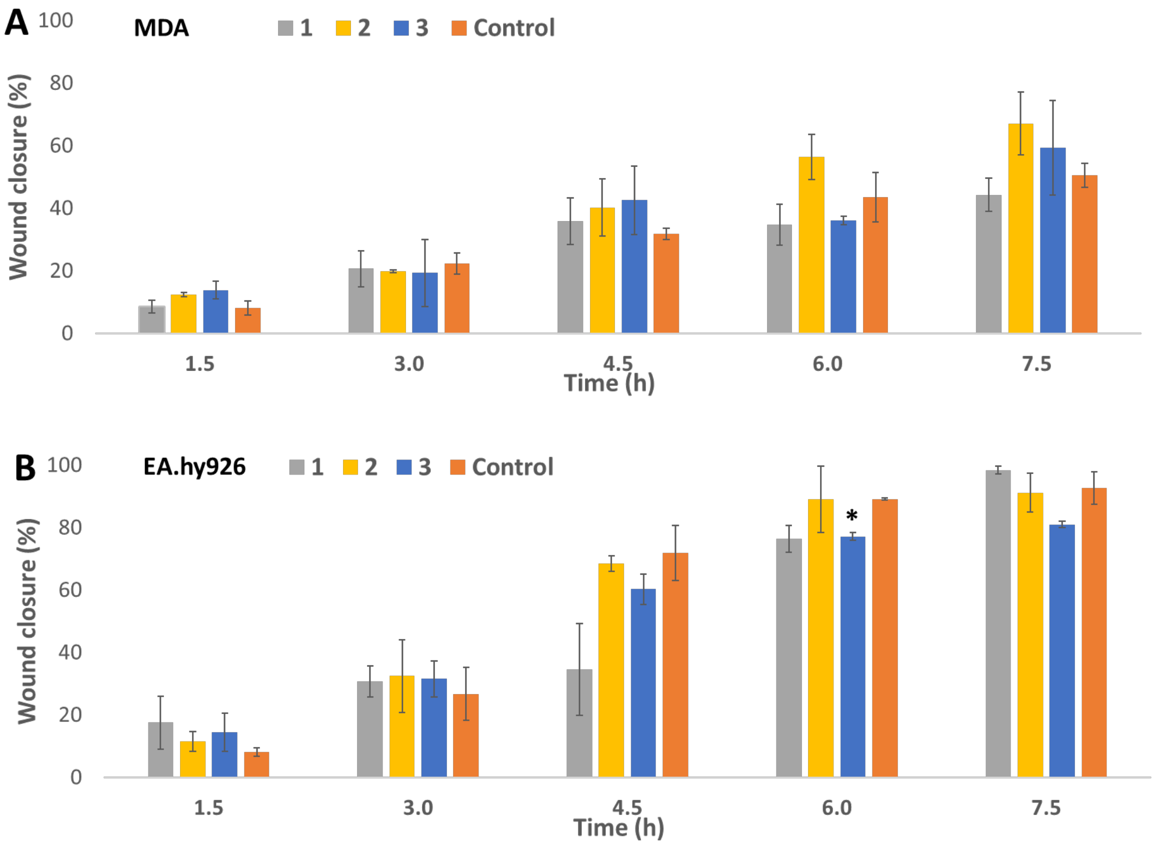

Figure S17. Wound closure rate of MDA cells (A) and EA.hy926 cells (B) after labelling with DBCO-probes **1-3** and compared to untreated control. Statistical significance versus Control group using unpaired Student's t-test: * p ≤ 0.05, ** p ≤ 0.01, *** p ≤ 0.001.

Table S7. Average wound area and wound closure percentage of HeLa cells.

	Control		ITag-1		ITag-2		ITag-3	
Time (h)	Wound closure (%)	Standard error of mean	Wound closure (%)	Standard error of mean	Wound closure (%)	Standard error of mean	Wound closure (%)	Standard error of mean
0	0.00	0.00	0.00	0.00	0.00	0.00	0.00	0.00
1.5	13.63	1.18	13.63	1.18	6.83	1.17	19.42	7.02
3	21.12	1.38	21.12	1.38	19.17	5.57	28.43	6.23
4.5	29.97	3.58	29.97	3.58	39.86	9.27	47.02	10.12
6	65.59	4.64	65.59	4.64	60.10	12.73	64.98	9.71
7.5	70.81	3.05	70.81	3.05	55.12	1.25	72.45	12.13
9	85.29	5.98	85.29	5.98	69.11	1.22	72.74	11.21

Table S8. Average wound area and wound closure percentage of MDA cells.

	Control		ITag-1		ITag-2		ITag-3	
Time (h)	Wound closure (%)	Standard error of mean	Wound closure (%)	Standard error of mean	Wound closure (%)	Standard error of mean	Wound closure (%)	Standard error of mean
0	0,00	0.00	0.00	0.00	0.00	0.00	0.00	0.00
1.5	8.02	3.99	8.49	3.58	12.28	1.01	13.77	5.05
3	22.24	5.71	20.58	10.08	19.85	0.61	19.27	18.52
4.5	31.76	3.12	35.76	13.03	40.16	12.93	33.13	19.11
6	43.37	13.64	34.57	11.30	56.26	10.26	45.31	2.27
7.5	50.415	6.50	44.21	9.09	67.02	14.07	59.27	26.07

Table S9. Average wound area and wound closure percentage of EA.hy926 cells.

	Control		ITag-1		ITag-2		ITag-3	
Time (h)	Wound closure (%)	Standard error of mean	Wound closure (%)	Standard error of mean	Wound closure (%)	Standard error of mean	Wound closure (%)	Standard error of mean
0	0.00	0.00	0.00	0.00	0.00	0.00	0.00	0.00
1.5	8.00	1.40	17.48	8.45	11.39	3.20	14.39	6.11
3	26.64	8.49	30.57	4.97	32.43	11.67	31.46	5.79
4.5	71.79	8.79	34.53	14.70	68.48	2.46	60.23	4.87
6	89.05	0.28	76.31	4.29	89.02	10.67	77.08	1.26
7.5	92.68	7.32	98.33	1.67	91.11	8.89	80.88	1.45

Table S10. Average wound area and wound closure percentage of Sialidase-treated HeLa cells.

	Control		ITag-1	
Time (h)	Wound closure (%)	Standard error of mean	Wound closure (%)	Standard error of mean
0	0.00	0.00	0.00	0.00
1.5	18.09	0.64	22.64	4.59
3	43.62	0.57	46.91	2.43
4.5	73.37	3.57	63.94	2.85
6	81.86	2.35	87.47	1.67
7.5	90.04	3.65	88.49	3.39

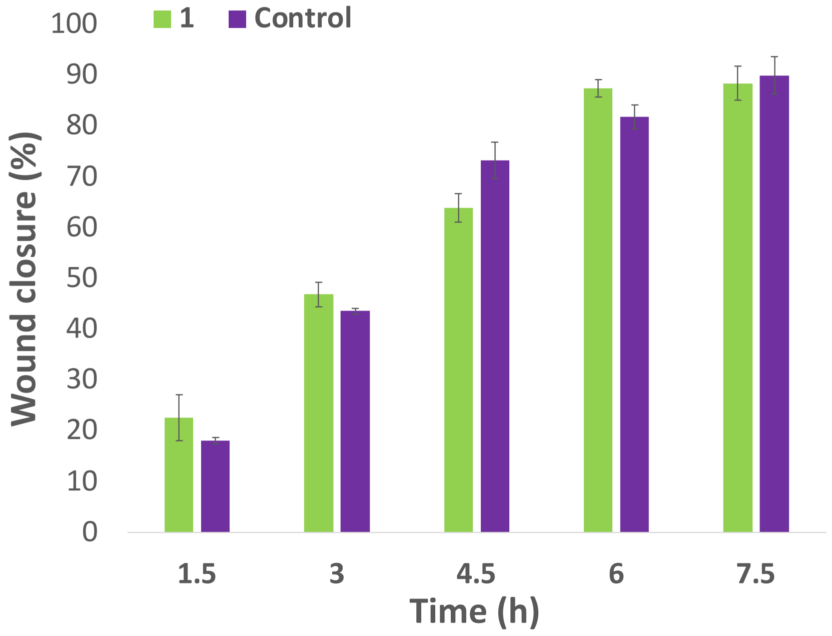

Figure S18. Would closure rate of neuraminidase-treated HeLa cells. White scale bars = 200 µM distance. Snapshots are taken at the timeframe indicated on column A. ns: $p > 0.05$, *: $p < 0.05$, **:

3. Supplementary references

[1] D. C. Forbes, S. A. Patrawala, K. L. T. Tran, *Organometallics* **2006**, *25*, 2693-2695.
